# Angioscopic Observation After Coronary Angioplasty for Chronic Coronary Occlusion Comparison With Severe Stenotic Lesion

**DOI:** 10.1155/DTE.7.7

**Published:** 2000

**Authors:** Takayoshi Adachi, Atsushi Hirayama, Masanori Asakura, Osamu Yamaguchi, Yasunori Ueda, Tsunehiko Kuzuya, Masatsugu Hori, Kazuhisa Kodama

**Affiliations:** 1 Department of Cardiology Osaka South National Hospital Osaka Japan; 2 Cardiovascular Division Osaka Police Hospital Osaka Japan; 3 Division of Cardiology Department of Internal Medicine and Therapeutics Osaka University Graduate School of Medicine Suita Japan

## Abstract

*Objectives* To clarify the underlying mechanism for the high restenosis rate after the coronary angioplasty for the chronic total occlusion by using the coronary angioscope.

*Background* Coronary angioplasty for the chronic total occlusion is associated with higher restenosis rate than for highly stenotic lesion. However, the difference in the restenosis rate has not been discussed from the angioscopic observation.

*Methods and Results* The lesion morphology after coronary intervention were classified into 4 grade (Grade 0 = no intimal flap; Grade 1 = intimal flap without protrusion; Grade 2 = Intimal flap with protrusion not occlusive; Grade 3 = protruding intimal flaps with occlusion of the vessel lumen). Coronary angioscopic observation was performed in 46 patients with stable angina. Most of the lesion morphology after angioplasty in 13 patients with chronic total occlusion was grade 3. On the other hand, none of grade 3 was observed in 36 patients with severe coronary stenosis.

*Conclusion* The various protrusions into the lumen shown by the angioscope might be a reason for higher restenosis and reocclusion rates compared with those after the angioplasty for the severe stenotic lesion.


*Diagnostic and Therapeutic Endoscopy*, Vol. 7, pp. 7–14
Reprints available directly from the publisher
Photocopying permitted by license only

© 2000 OPA (Overseas Publishers Association) N.V.
Published by license under
the Harwood Academic Publishers imprint,
part of The Gordon and Breach Publishing Group.
Printed in Malaysia.

### Angioscopic Observation after Coronary Angioplasty for Chronic Coronary Occlusion Comparison with Severe Stenotic Lesion

TAKAYOSHI ADACHI[a], ATSUSHI HIRAYAMA[b], MASANORI ASAKURA[c], OSAMU YAMAGUCHI[b], YASUNORI UEDA[b], TSUNEHIKO KUZUYA[c], MASATSUGU HORI[c] and KAZUHISA KODAMA[b,*]

[a]*Department of Cardiology, Osaka South National Hospital;* [b]*Cardiovascular Division, Osaka Police Hospital, Osaka, Japan;* [c]*Division of Cardiology, Department of Internal Medicine and Therapeutics, Osaka University Graduate School of Medicine, Suita, Japan*

*(Received 27 April 2000; Revised 12 June 2000; In final form 12 June 2000)*

***Objectives*** **To clarify the underlying mechanism for the high restenosis rate after the coronary angioplasty for the chronic total occlusion by using the coronary angioscope.**

***Background*** **Coronary angioplasty for the chronic total occlusion is associated with higher restenosis rate than for highly stenotic lesion. However, the difference in the restenosis rate has not been discussed from the angioscopic observation.**

***Methods and Results*** **The lesion morphology after coronary intervention were classified into 4 grade (Grade 0 = no intimal flap; Grade 1 = intimal flap without protrusion; Grade 2 = Intimal flap with protrusion not occlusive; Grade 3 = protruding intimal flaps with occlusion of the vessel lumen). Coronary angioscopic observation was performed in 46 patients with stable angina. Most of the lesion morphology after angioplasty in 13 patients with chronic total occlusion was grade 3. On the other hand, none of grade 3 was observed in 36 patients with severe coronary stenosis.**

***Conclusion*** **The various protrusions into the lumen shown by the angioscope might be a reason for higher restenosis and reocclusion rates compared with those after the angioplasty for the severe stenotic lesion.**

*Keywords*: Angioplasty, Chronic total occlusion, Coronary angioscope, Stent

### INTRODUCTION

Percutaneous transluminal coronary angioplasty has become a standard treatment for the patients with coronary heart disease. With the substantial advances in devices and improvements in operators' skill, coronary angioplasty has been applied to the various type of coronary morphology, so called complex lesions. One of the complex lesions is the chronic total occlusion. Since the first study

* Corresponding author. Tel.: 81-6-6771-6051. Fax: 81-6-6775-2845.

cohort was reported in 1982 [1], many studies dealing with chronic total occlusions has been reported [2]. The beneficial effects of successful angioplasty for the chronic total occlusion has been reported: Anginal state often improves after successful angioplasty; left ventricular functions may improve [3–5]; and subsequent referral for coronary bypass graft surgery is uncommon [6–8]. In spite of these beneficial effects, two major problems are existed. One is the initial success rate, which has reached over 70% in recent reports [2,9]. Selection of the lesions for more favorable outcome and newer devices may improve the initial success rate [10]. Another problem is the very high restenosis rate following the successful recanalization. A restenosis rate of 44 to 77% limits the long-term benefit of angioplasty [11], however the reason for this high restenosis rate has not been elucidated.

Coronary angioscope is a tool for the intracoronary imaging and would be useful to observe characteristics of the lesions before and after coronary interventions to evaluate the effects of the interventions. Our coronary angioscopic catheter is 0.75 mm in outer diameter with a fiber containing 6000 pixels, which has power to observe clearly the neointimal coverage of the stent [12,13] or the lesion characteristics in patients with acute coronary syndrome received reperfusion therapy [14].

We observes the chronically total lesions with angioscope after successful angioplasty may help to understand the high restenosis rate. In this point of view, we angioscopically studied the lesion morphology with chronic total occlusion after successful intervention and compared with that of after successful the high grade stenoses and discussed the mechanism for the high rate of restenosis for the chronic total occlusion.

### METHODS

### Patients Selection

Of patients with stable angina treated angioplasty from January to December 1996, 49 patients were enrolled in this study; Thirteen patients with successful angioplasty for chronic total occlusion (CTO group) and 36 patients with successful angioplasty for severe stenosis lesion (non-CTO group). None of the patients within one month after acute myocardial infarction and with poor left ventricular function were enrolled in this study. If the angioscopic images obtained after intervention, patients were excluded. This study protocol was approved by the Osaka Police Hospital Ethical Committee.

### Angioscopic Procedures and Evaluations

After obtaining the written informed consent, coronary angiography and intervention were performed according to standard procedure by the femoral approach. Angiographic success of the dilatation was achieved when the stenosis diameter after the balloon was $< 50\%$. If the angiographic appearance of the target lesion was suboptimal by coronary angiograms, further large size balloon was used until the satisfactory results were obtained. After the completion of successful dilatation, angioscopic observation were made. We used the angioscope MC-800E (Nihon Kohden) and the optic fiber AS-003 (Nihon Kohden). The angioscopic observations were made while the blood was cleared away from the view by the injection of 3% dextran-40 as described previously [12]. We examined the culprit lesions treated by intervention and evaluated the lesion morphology. The lesion morphology were classified into 4 grades as shown in Fig. 1; grade 0 is lesion with no flap, grade 1 is lesion with the intimal flap without protrusion, grade 2 is lesion with protruding flaps without occlusion of the vessel lumen, and grade 3 is lesion with protruding flaps occluding the vessel lumen. The presence or absence of yellow plaque and thrombus at culprit lesion after angioplasty was also evaluated by the angioscope. The angioscopic images were reviewed by a specialist who was unaware of the angiographic lesion characteristics and patient's background.

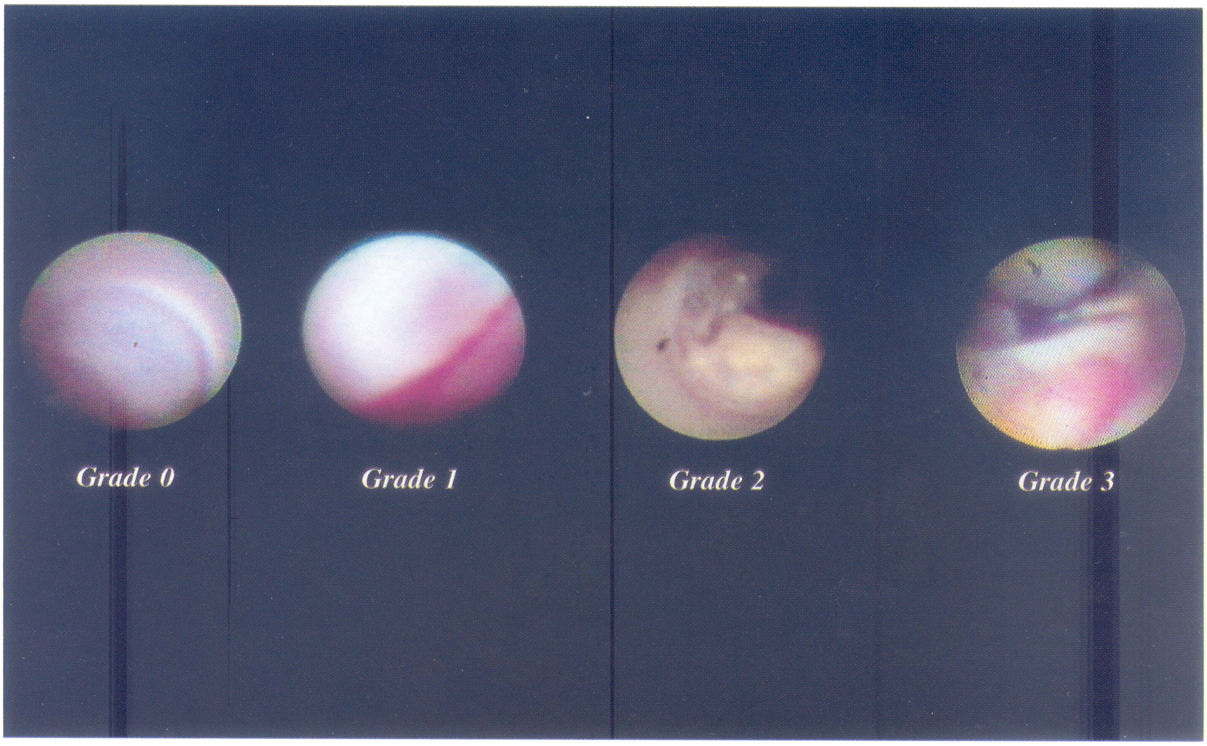

FIGURE 1 Classification of lesion morphology observed by angioscope. Grade 0 = no intimal flap; Grade 1 = intimal flap without protrusion; Grade 2 = intimal flap with protrusion not occlusive; Grade 3 = protruding intimal flaps with occlusion of the vessel lumen.

### Statistics

All data were presented as mean ± SD. For the analysis of the difference between two groups, the two-tailed Student *t* test was used in case of standard deviation. Categorical data were compared by the chi-square test. A statistical probability of $p < 0.05$ was considered to be significant.

### RESULTS

### Patients Characteristics

The baseline characteristics were shown in the Table I. There were no differences between CTO and non-CTO group in terms of age, gender and target vessel distribution. The incidence of previous myocardial infarction was significant higher in CTO group than in non-CTO group.

### Angioscopic Observation

Fig. 2 shows the representative angioscopic findings of non-CTO group. Yellow plaque was observed at the lesion and intimal flap with protrusion was also observed and its lesion morphology was classified into grade 2. Fig. 3 showed the representative angioscopic findings of CTO group. The yellow plaque with thrombus was observed at the lesion and tremendous amounts of intimal flap occluding the lumen with wire was observed. The lesion morphology was grade 3. The distribution of lesion morphology were shown in Fig. 4. After intervention, grade 3 was 62% in CTO group, however grade 3 was observed in only 1

TABLE I Base line characteristics between CTO group and non-CTO group

| | CTO group | Non-CTO group | $p$ value |
|---|---|---|---|
| Age (yrs) | 55 ± 9 | 60 ± 11 | ns |
| Sex (male/female) | 11/2 | 30/6 | ns |
| Previuos MI (%) | 84.6% | 52.8% | ns |
| Target artery (LAD/LCx/RCA) | 9/0/4 | 21/3/12 | ns |

MI: myocardial infarction, LAD: left ascending artery, LCx: Left circumfex artery, RCA: right coronary artery.

patient (3%). On the other hand, grade 0 was 24% in the non-CTO group, nevertheless grade 0 was observed in none of patients with CTO group. Thus, the significant distribution differences in lesion morphology between CTO and non-CTO group ($p < 0.05$). The yellow plaque was observed in all patients with CTO group and 83.3% in non-CTO group, but the difference was not significant. Thrombus was observed more frequently in non-CTO group (47.2%) rather than CTO group (30.8%), but the difference was not significant (Fig. 5).

### DISCUSSION

Coronary angioscope revealed a terrible intra-coronary observation after the revascularization

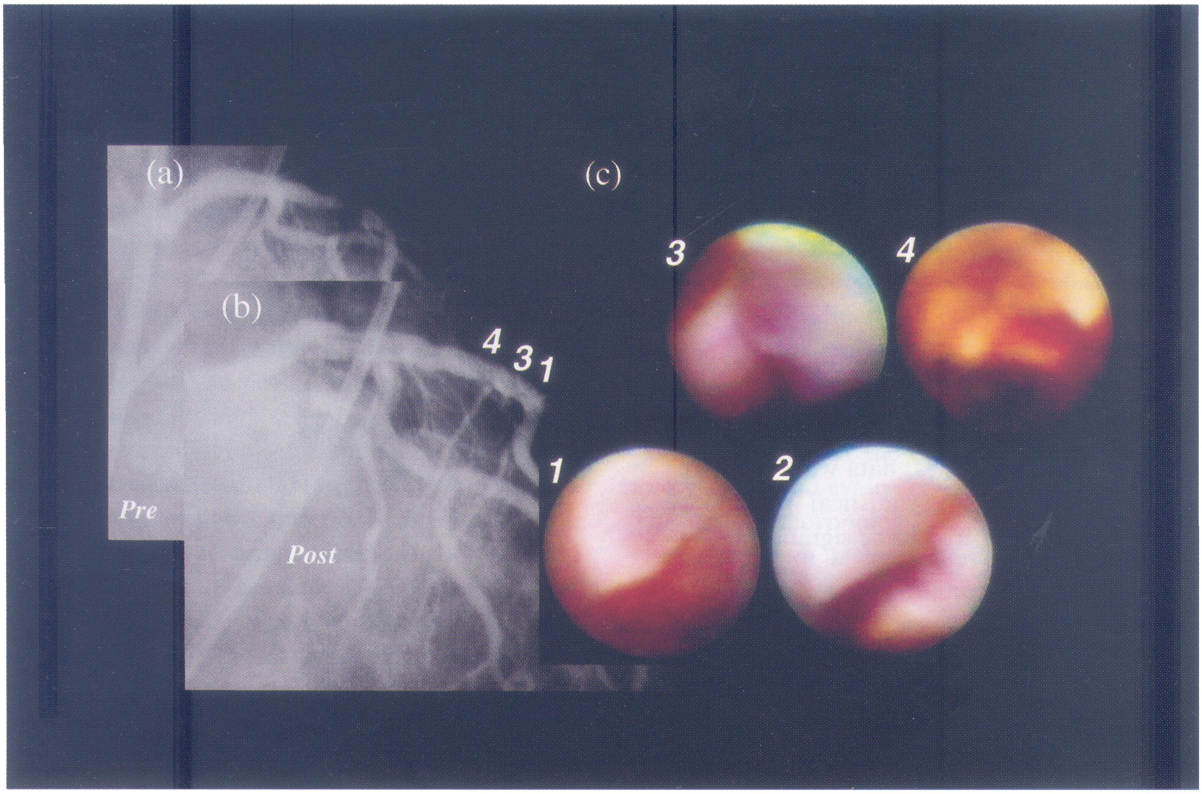

FIGURE 2 Representative angioscopic images after angioplasty for severe stenotic lesion (non-CTO group). Coronary angiograms pre (a), post intervention (b) and the angioscopic images after intervention (c) were shown. The number of each angioscopic image corresponds to that of the location shown in the coronary angiogram. Yellow plaque was observed at the culprit lesion and non-occlusive intimal flaps were observed.

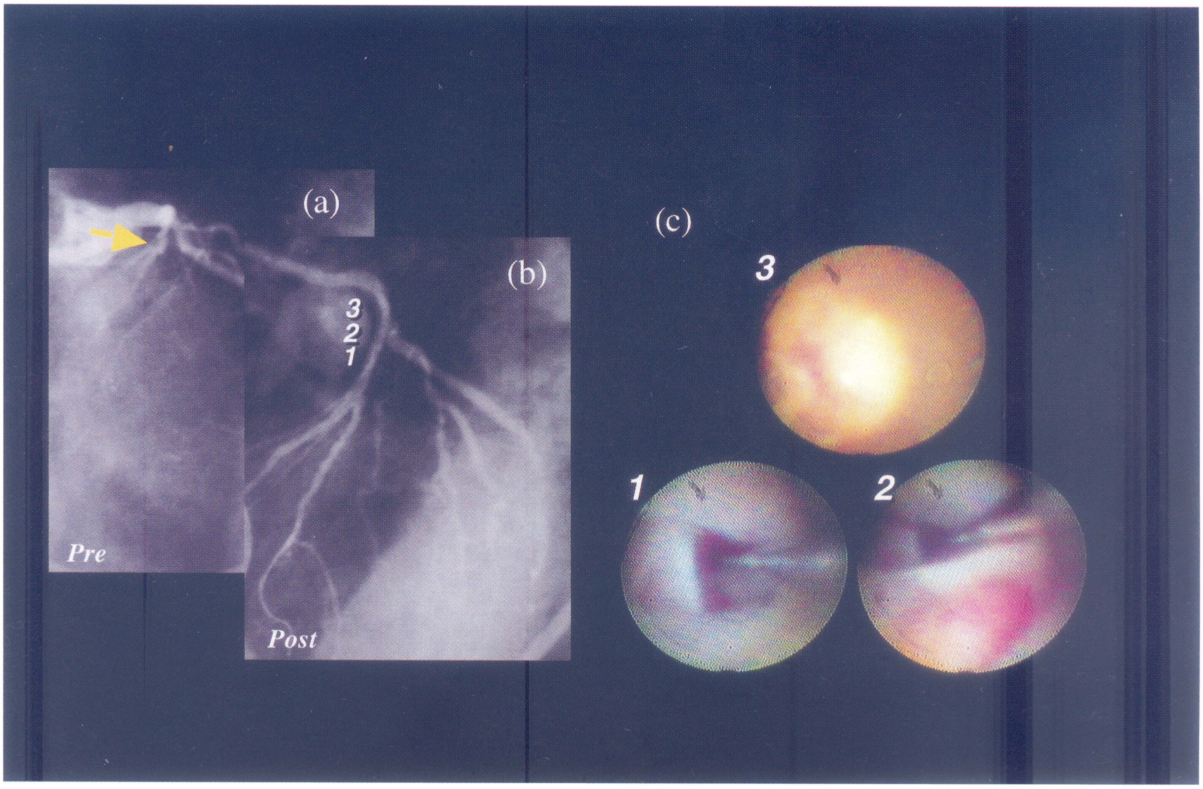

FIGURE 3 Representative angioscopic images after angioplasty for chronic total occlusion (CTO group). Coronary angiograms pre (a), post intervention (b) and the angioscopic images after intervention (c) were shown. The number of each angioscopic image corresponds to that of the location shown in the coronary angiogram. Yellow plaque and white thrombus was observed at the culprit lesion and occlusive protrusions were observed around the guide wire.

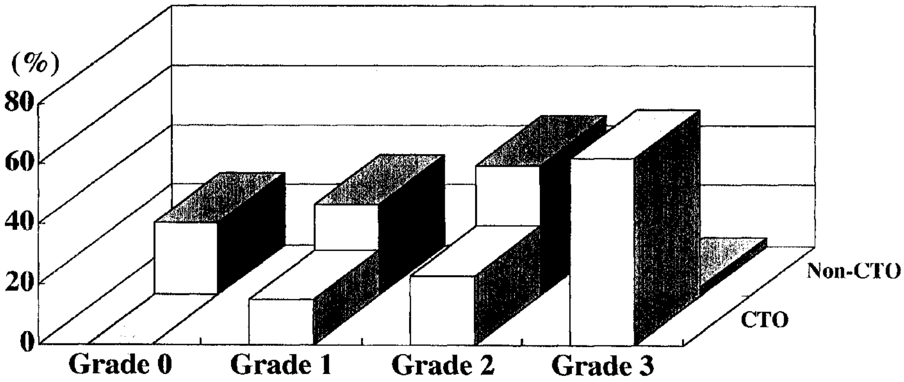

FIGURE 4 Distribution of the lesion morphology evaluated by angioscope after angioplasty between CTO and non-CTO group.

of the chronic total occlusion, that is, large intimal flaps or the thrombus with various lesions at the culprit site protrude from the vessel wall and occupy the vessel lumen, even if angiography showed the enough vessel lumen after the balloon angioplasty as described before [15]. On the other hand, angioscope after the successful angioplasty for the severe stenotic lesion showed also flaps, but the amounts and the degree of protrusions were less than after chronic total occlusion. These protruding lesions might disturb the coronary flow and causes the thrombus formation. Thrombus once formed at the intervention lesion contribute the restenosis by itself as well as enhance the neointimal hyperplasia [16], the other cause of restenosis [17]. Thus, the various protrusions into the lumen shown by the angioscope might be a reason for higher restenosis and reocclusion rates compared with those after the angioplasty for the severe stenotic lesion.

Recently, coronary artery stenting has emerged as a valuable therapeutic strategy for the management of chronic total occlusion [18–20]. Intracoronary insertion of the Palmaz-Shatz stent or Wiktor stent after successful angioplasty of chronic total occlusion has been associated with favorable restenosis and reocclusion rate, improved short- and long-term outcome [21, 22]. Our angioscopic observation showed the suppression of the occlusive protruding intimal flaps or thrombus and keep the lumen open as shown in Fig. 6. The preventive mechanism for the restenosis of stent is explained by the prevention of chronic vessel shrinkage [23], However, these compressions of protruding materials to the vessel wall might be the another mechanism for the prevention in the case of chronic total occlusion.

All patients with chronic total occlusion in our study suffered from myocardial infarction previously. As it is well known that acute myocardial infarction caused by the occlusive thrombus formation following plaque rupture, the total lesion suspected to have rich thrombus. The angioscopic observation showed that fewer incidence of thrombus in the CTO group compared with non-CTO group. Total occlusion comprises athrosclerotic plaque and a single or multiple layers of clot formed by repeat injury repair process [2]. Angioscope can recognize fresh clots as a thrombus, however as the older and more fibrosed clot were present in the lesion with total occlusion, angioscope cannot detect the thrombus.

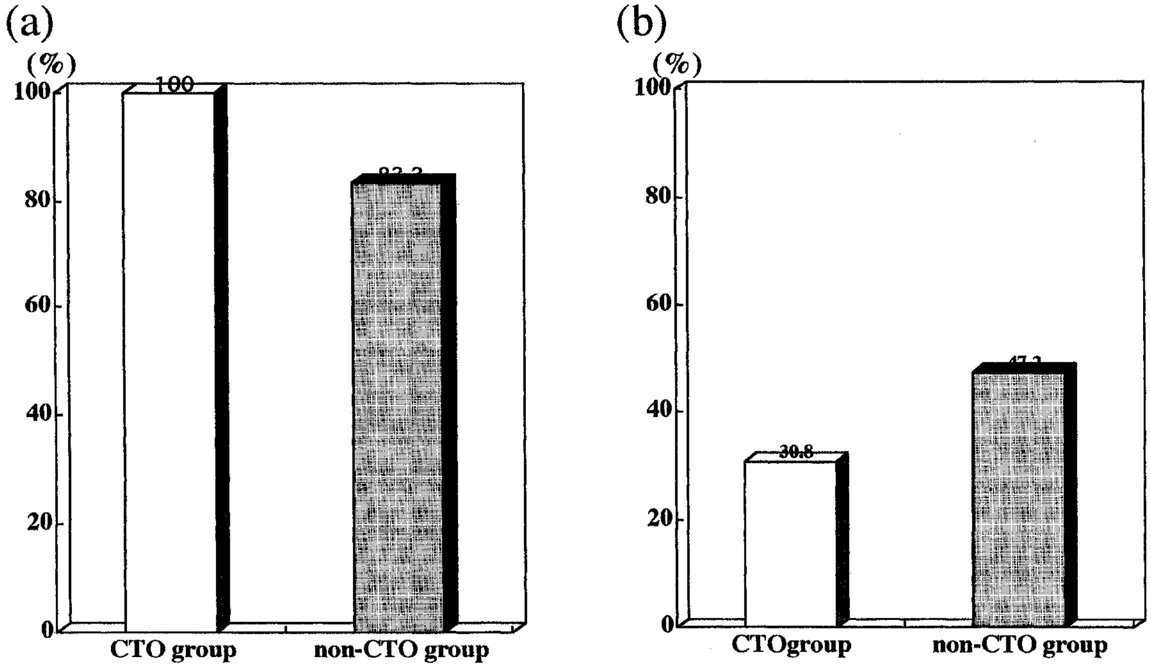

FIGURE 5 Incidence of yellow plaques (a) and thrombus (b) at the culprit lesion in CTO and non-CTO group.

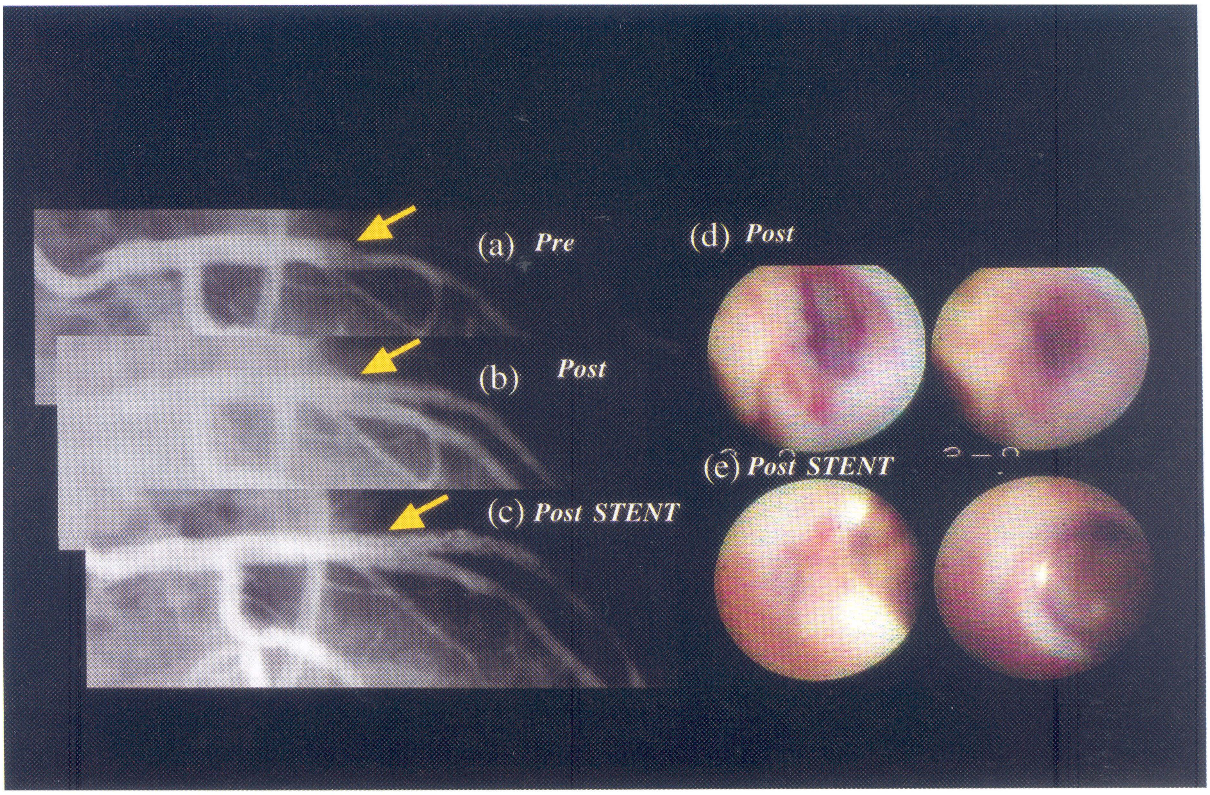

FIGURE 6 The compression of occlusive protrusions after angioplasty after chronic total occlusion by stent. Coronary angiograms pre (a), post (b) and after stenting (c) were shown. Angioscopic images after angioplasty and stenting were shown in (d) and (e), respectively.

Furthermore, histologic findings depicted that there were loose fibrous tissue was dispersed in the total occlusion site [24], which means less thrombus. The culprit lesions with high stenotic grade showed yellow plaque in 80% of patients and thrombus formation in half of them. The incidence of the yellow plaques a little bit higher than the previous reports indicating that yellow plaques were detected in the target vessel of angioplasty in about 60% of patients with stable effort angina [25]. It may be explained that the high incidence of yellow plaque in this study patients with severe stenotic lesions were selected among the patients with stable effort angina subjected to angioplasty. This also may explain the high incidence of thrombus observed by angioscope in patients with non-CTO group.

### *References*

[1] Savage, R., Holiman, J., Gruentzig, A.R., King, S., Douglas, J. and Tankersley, R. Can percutaneous transluminal coronary angioplasty be performed in patients with total occlusion? *Circulation* 1982; **66**: Supple II:II–330.

[2] Puma, J.A., Sketch, M.H., Jr., Tcheng, J.E., Harrington, R.A., Phillips, H.R., Stack, R.S. *et al.* Percutaneous revascularization of chronic coronary occlusions: an overview. *J. Am. Coll. Cardiol.* 1995; **26**(1): 1–11.

[3] Stewart, J.T., Denne, L., Bowker, T.J., Mulcahy, D.A., Williams, M.G., Buller, N.P. *et al.* Percutaneous transluminal coronary angioplasty in chronic coronary artery occlusion. *J. Am. Coll. Cardiol.* 1993; **21**(6): 1371–1376.

[4] Ruocco, N.A., Jr., Ring, M.E., Holubkov, R., Jacobs, A.K., Detre, K.M. and Faxon, D.P. Results of coronary angioplasty of chronic total occlusions (the National Heart, Lung, and Blood Institute 1985–1986 Percutaneous Transluminal Angioplasty Registry). *Am. J. Cardiol.* 1992; **69**(1): 69–76.

[5] Bell, M.R., Berger, P.B., Bresnahan, J.F., Reeder, G.S., Bailey, K.R. and Holmes, D.R. Jr., Initial and long-term outcome of 354 patients after coronary balloon angioplasty of total coronary artery occlusions [see comments]. *Circulation* 1992; **85**(3): 1003–1011.

[6] Ivanhoe, R.J., Weintraub, W.S., Douglas , J.S. Jr., Lembo, N.J., Furman, M., Gershony, G. *et al.* Percutaneous transluminal coronary angioplasty of chronic total occlusions. Primary success, restenosis, and long-term clinical follow-up [see comments]. *Circulation* 1992; **85**(1): 106–115.

[7] Warren, R.J., Black, A.J., Valentine, P.A., Manolas, E.G. and Hunt, D. Coronary angioplasty for chronic total occlusion reduces the need for subsequent coronary bypass surgery. *Am. Heart J.* 1990; **120**(2): 270–274.

[8] Finci, L., Meier, B., Favre, J., Righetti, A. and Rutishauser, W. Long-term results of successful and failed angioplasty for chronic total coronary arterial occlusion. *Am. J. Cardiol.* 1990; **66**(7): 660–662.

[9] Kinoshita, I., Katoh, O., Nariyama, J., Otsuji, S., Tateyama, H., Kobayashi, T. *et al.* Coronary angioplasty of chronic total occlusions with bridging collateral vessels: immediate and follow-up outcome from a large single-center experience. *J. Am. Coll. Cardiol.* 1995; **26**(2): 409–415.

[10] Rees, M.R., Michalis, L.K., Pappa, E.C., Loukas, S., Goudevenos, J.A. and Sideris, D.A. The use of soft and flexible guidewires in the treatment of chronic total coronary occlusions by activated guidewire angioplasty. *Br. J. Radiol.* 1999; **72**(854): 162–167.

[11] Ellis, S.G., Shaw, R.E., Gershony, G., Thomas, R., Roubin, G.S., Douglas, J.S. Jr., *et al.* Risk factors, time course and treatment effect for restenosis after successful percutaneous transluminal coronary angioplasty of chronic total occlusion. *Am. J. Cardiol.* 1989; **63**(13): 897–901.

[12] Ueda, Y., Nanto, S., Komamura, K. and Kodama, K. Neointimal coverage of stents in human coronary arteries observed by angioscopy. *J. Am. Coll. Cardiol.* 1994; **23**(2): 341–346.

[13] Asakura, M., Ueda, Y., Nanto, S., Hirayama, A., Adachi, T., Kitakaze, M. *et al.* Remodeling of in-stent neointima, which became thinner and transparent over 3 years: serial angiographic and angioscopic follow-up. *Circulation* 1998; **97**(20): 2003–2006.

[14] Ueda, Y., Asakura, Y., Hirayama, A., Komamura, K., Hori, H. and Kodama, K. Intracoronary morphology of culprit lesions after reperfusion in acute myocardial infarction: Serial angioscopic observations. *J. Am. Coll. Cardiol.* 1996; **27**: 606–610.

[15] Alfonso, F., Goicolea, J., Hernamdez, R., Gomcalves, M. *et al.* Angioscopic findings during coronary angioplasty of coronary occlusion. *J. Am. Coll. Cardiol.* 1995; **26**: 135–141.

[16] Preisack, M.B. and Karsch, K.R. The paradigm of restenosis following percutaneous transluminal coronary angioplasty. *Eur. Heart. J.* 1993; **14** Suppl I: 187–192.

[17] Forrester, J.S., Fishbein, M., Helfant, R. and Fagin, J. A paradigm for restenosis based on cell biology: clues for the development of new preventive therapies. *J. Am. Coll. Cardiol.* 1991; **17**(3): 758–769.

[18] Medina, A., Melian, F., Suarez de Lezo, J., Pan, M., Romero, M., Hernandez, E. *et al.* Effectiveness of coronary stenting for the treatment of chronic total occlusion in angina pectoris. *Am. J. Cardiol.* 1994; **73**(16): 1222–1224.

[19] Mori, M., Kurogane, H., Hayashi, T., Yasaka, Y., Ohta, S., Kajiya, T. *et al.* Comparison of results of intracoronary implantation of the Plamaz- Schatz stent with conventional balloon angioplasty in chronic total coronary arterial occlusion. *Am.. J. Cardiol.* 1996; **78**(9): 985–989.

[20] Ozaki, Y., Violaris, A.G., Hamburger, J., Melkert, R., Foley, D., Keane, D. *et al.* Short- and long-term clinical and quantitative angiographic results with the new, less shortening Wallstent for vessel reconstruction in chronic total occlusion: a quantitative angiographic study. *J. Am. Coll. Cardiol.* 1996; **28**(2): 354–360.

[21] Anzuini, A., Rosanio, S., Legrand, V., Tocchi, M., Coppi, R., Bonnier, H. *et al.* Wiktor stent for treatment of chronic total coronary artery occlusions: short- and long-term clinical and angiographic results from a large multicenter experience. *J. Am. Coll. Cardiol.* 1998; **31**(2): 281–288.

[22] Hoher, M., Wohrle, J., Grebe, O.C., Kochs, M., Osterhues, H.H., Hombach, V. *et al.* A randomized trial of elective stenting after balloon recanalization of chronic total occlusions. *J. Am. Coll. Cardiol.* 1999; **34**(3): 722–729.

[23] Mintz, G.S., Popma, J.J., Pichard, A.D., Kent, K.M., Satler, L.F., Hong, M.K. *et al.* Intravascular Ultrasound Assessment of the Mechanisms and Predictors of Restenosis Following Coronary Angioplasty. *J. Invasive Cardiol.* 1997; **9**(4): 303–314.

[24] Katsuragawa, M., Fujiwara, H., Miyamae, M. and Sasayama, S. Histologic studies in percutaneous transluminal coronary angioplasty for chronic total occlusion: comparison of tapering and abrupt types of occlusion and short and long occluded segments. *J. Am. Coll. Cardiol.* 1993; **21**(3): 604–611.

[25] Thieme, T., Wernecke, K.D., Meyer, R., Brandenstein, E., Habedank, D., Hinz, A. *et al.* Angioscopic evaluation of atherosclerotic plaques: validation by histomorphologic analysis and association with stable and unstable coronary syndromes. *J. Am. Coll. Cardiol.* 1996; **28**(1): 1–6.

